# Case report: life-threatening endocarditis in an adult with repaired congenital heart disease lost to follow-up

**DOI:** 10.1093/ehjcr/ytag284

**Published:** 2026-04-22

**Authors:** Ryan Michael Wilson, Zainab Arif, Lydia Sturridge

**Affiliations:** Department of General Medicine, Frimley Health NHS Foundation Trust, Portsmouth Road, Frimley, Camberley, Surrey GU16 7UJ, UK; Department of General Medicine, Frimley Health NHS Foundation Trust, Portsmouth Road, Frimley, Camberley, Surrey GU16 7UJ, UK; Department of General Medicine, Frimley Health NHS Foundation Trust, Portsmouth Road, Frimley, Camberley, Surrey GU16 7UJ, UK

**Keywords:** Infective endocarditis, Adult congenital heart disease, Delayed diagnosis, Transitional care, Systemic failure, Case report

## Abstract

**Background:**

Adults with repaired congenital heart disease remain vulnerable to complications such as valvular dysfunction, aortic dissection, and infective endocarditis. Loss to specialist follow-up and incomplete synthesis of early warning signs can delay diagnosis. This abstract highlights how such factors combined to produce a near-fatal presentation of endocarditis.

**Case summary:**

A 58-year-old man with childhood repair of aortic coarctation, otherwise previously healthy, presented 1 week after a dental filling without antibiotic prophylaxis. He developed fever, back pain, and hypoxia. Treated initially for pneumonia, he failed to improve. Progressively abnormal conduction, embolic renal lesions, and refractory sepsis eventually prompted echocardiography, which revealed a severely calcified bicuspid aortic valve with large vegetations and an ascending aortic abscess. He underwent emergency valve replacement and aortic root patch repair, followed by prolonged intravenous antibiotics and implantation of cardiac resynchronization therapy.

**Discussion:**

This case underscores three system-level failures that nearly proved fatal: loss to follow-up during the paediatric-to-adult transition, dismissal of a solitary positive blood culture, and incomplete synthesis of multisystem ‘red flags’. Vigilant lifelong surveillance in adults with congenital heart disease, coupled with a low threshold for endocarditis work-up, including a point-of-care ultrasound in the acute setting in septic patients with structural heart lesions, is essential to improve outcomes, as well as appropriate dental prophylaxis in criteria-met adult congenital heart disease patients.

Learning points

- Adults with repaired congenital heart disease remain at lifelong risk of endocarditis and require sustained specialist follow-up.
- Any positive blood culture in a cardiac risk patient should prompt full investigation rather than dismissal as contamination.
- Red flags, such as conduction changes, emboli, or unexplained sepsis, must trigger early echocardiography to avoid diagnostic delay.

## Introduction

Adults with repaired congenital heart disease (ACHD) remain at lifelong risk of late complications, including infective endocarditis, yet gaps in surveillance, and fragmented transitions from paediatric to adult care frequently expose patients to avoidable harm. We present the case of a 58-year-old man with repaired aortic coarctation who developed fulminant endocarditis complicated by an ascending-aortic abscess, complete heart block, and septic emboli following recent dental work. Despite initial misdiagnosis and delayed recognition, urgent surgery, and multidisciplinary input secured recovery. This case highlights three system-level failures—loss to follow-up, dismissal of a positive blood culture, and missed early red flags—that nearly proved fatal. It reinforces the need for vigilant long-term surveillance, early echocardiography in septic ACHD patients, and a pragmatic approach to dental prophylaxis in high-risk groups, aligning with current ESC recommendations.

## Summary figure

The summary flowchart of the patients’ admission

**Figure:**
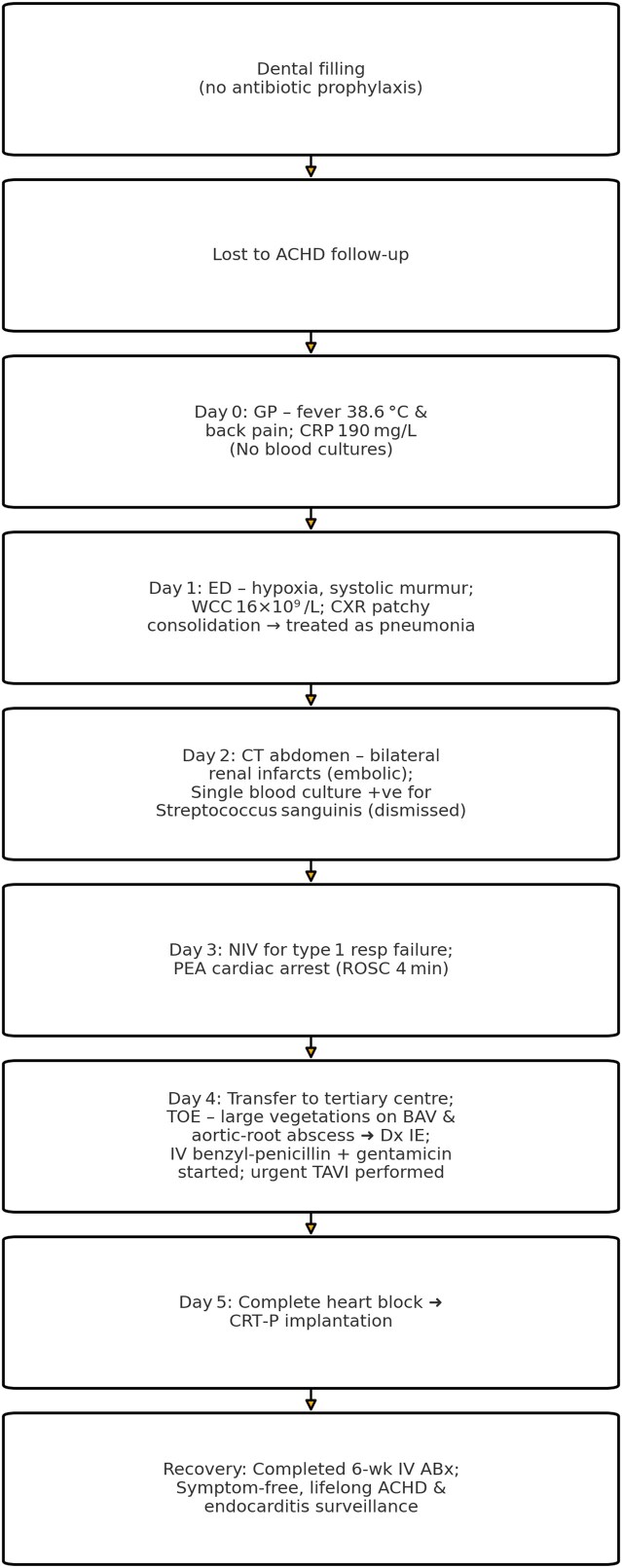


## Case description

### Medical history

A 58-year-old man had undergone childhood repair of aortic coarctation. He was otherwise previously healthy and had been lost to specialist cardiology follow-up for decades.

### Current illness

One week after a dental filling performed without antibiotic prophylaxis, he developed fever, rigours, drenching night sweats, diffuse myalgia, and bilateral flank pain. He presented to a district general hospital. On examination he had a long-standing systolic murmur but no peripheral stigmata of infective endocarditis.

### Clinical course

**Figure 1:**
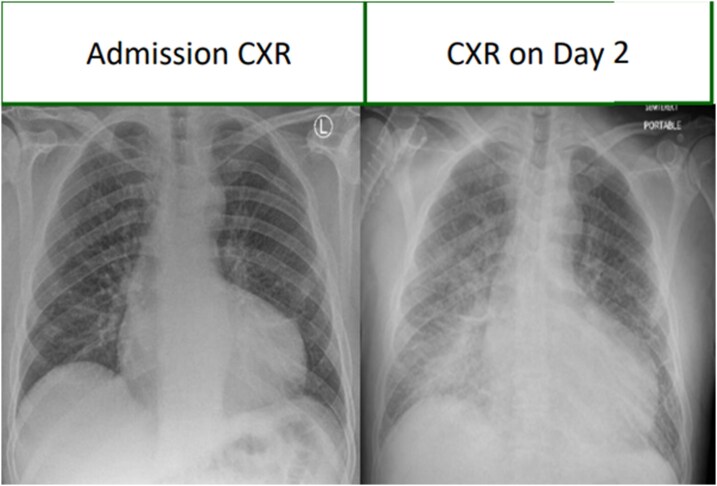
Chest radiograph demonstrating the development of consolidation.

**Figure 2:**
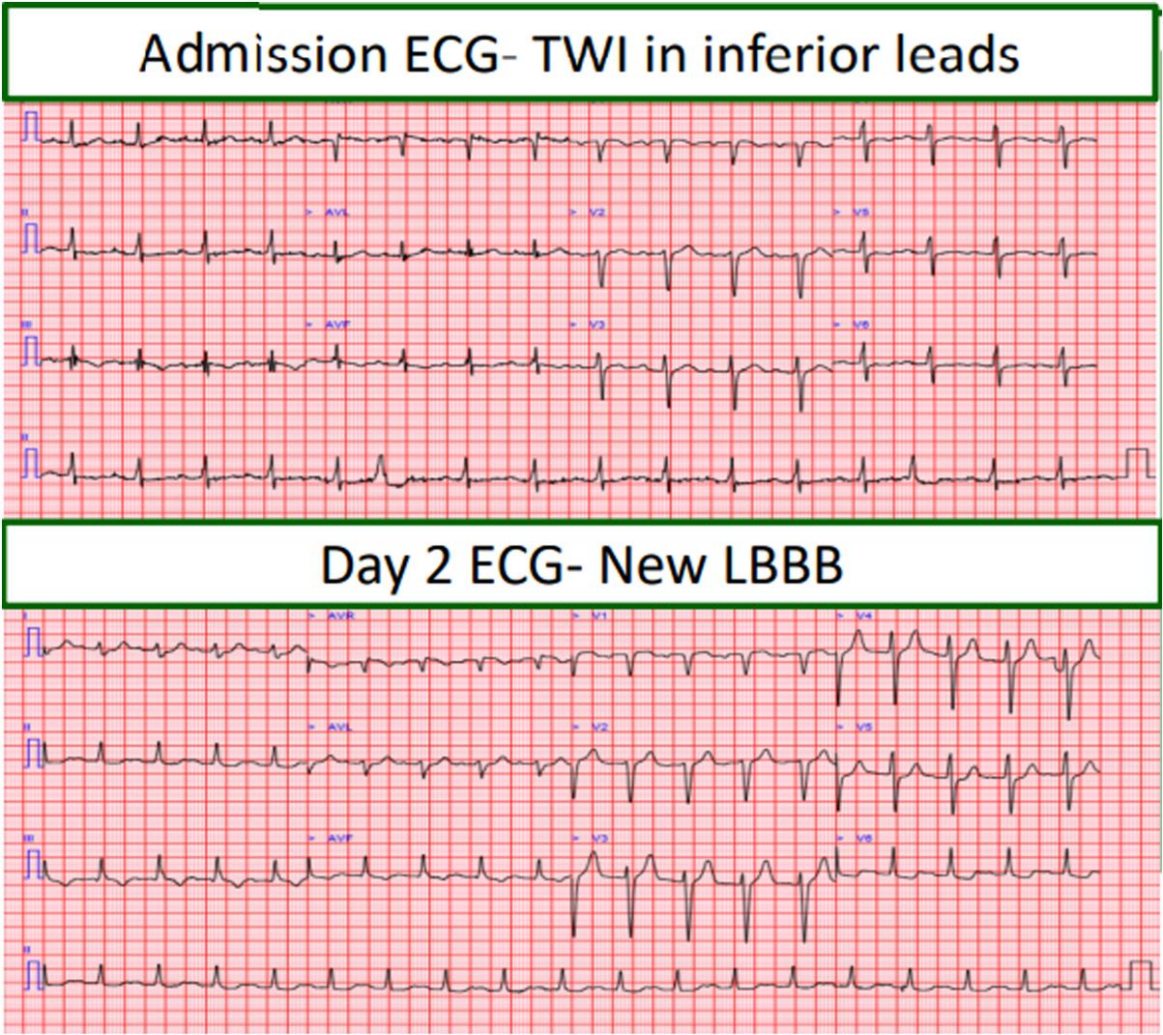
ECG demonstrating left bundle branch block development.

Initial investigations were non-specific. Empirical intravenous amoxicillin, metronidazole and gentamicin were started. Within 24 h, he developed worsening hypoxaemia; and both a chest radiograph and a CT chest showed right lower lobe consolidation (*Figure 1*), and antibiotics were escalated to piperacillin–tazobactam with gentamicin. Despite this, his condition deteriorated. CRP rose to 270 mg/L, troponin exceeded 10 000 ng/L, and ECGs demonstrated anterior T wave inversion followed by new left bundle branch block (*Figure 2*). Cardiology felt this represented Type II myocardial injury secondary to sepsis. A solitary positive blood culture with Gram-positive cocci was dismissed as contamination.

**Figure 3:**
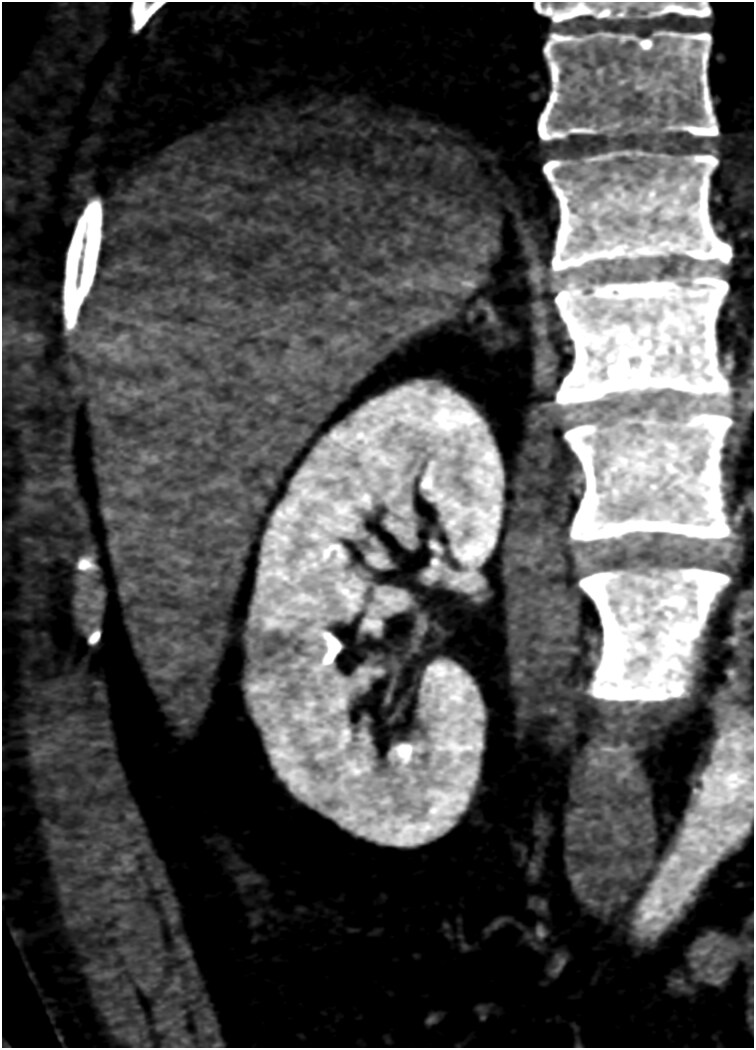
CT Abdo Pelvis demonstrating a right renal infarct.

Subsequent CT pulmonary angiography excluded pulmonary embolism but showed bilateral effusions and persistent consolidation. Abdominal CT identified a 16 mm hypodense right renal lesion, later confirmed as a septic embolus (*Figure 3*). An arterial blood gas demonstrated type I respiratory failure (PaO_2_ 7.75 kPa on supplemental oxygen). His hypoxaemia progressed, requiring CPAP (100% O_2_, PEEP 8 cm H_2_O) and ITU admission.

Shortly after intubation, he sustained a pulseless electrical activity arrest, regaining spontaneous circulation after 1 mg IV adrenaline and brief ALS. Repeat ECGs showed new ST-segment elevation. Blood cultures confirmed *Staphylococcus epidermidis*, leading to the addition of teicoplanin and later rifampicin. Bedside echocardiography revealed a heavily calcified bicuspid aortic valve with mobile vegetations and EF 20–30%. He suffered multiple brief runs of ventricular tachycardia, some causing transient loss of output but resolving spontaneously.

**Figure 4:**
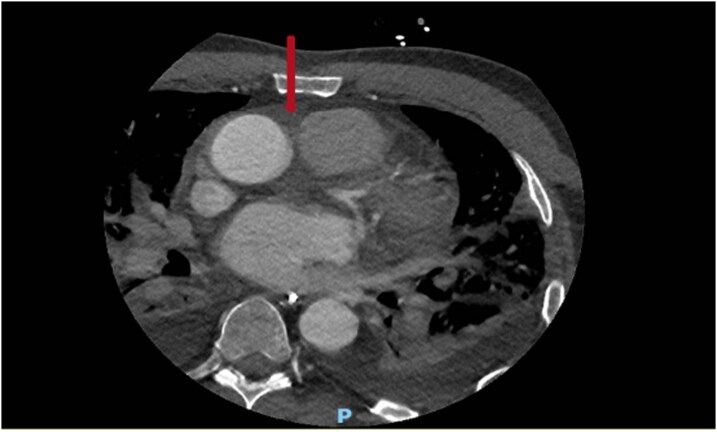
CT aorta demonstrating an aortic root abscess.

Transoesophageal echocardiography demonstrated valve perforation and an aortic root/ascending aortic abscess (*Figure 4*); CT aorta showed soft tissue thickening at the aortic root. He met ESC surgical criteria for urgent intervention.

At the tertiary centre, cultures grew *Staphylococcus hominis*, and antibiotics were changed to ceftriaxone and teicoplanin. Surgery comprised a 25 mm Resilia bioprosthetic aortic valve replacement, bovine pericardial patch repair of the aortic root abscess and ascending aorta, and drainage of bilateral pleural effusions. Post-operatively, he developed complete heart block requiring cardiac resynchronization therapy. Imaging confirmed clearance of infection, and he made a steady recovery, resuming full activity under routine cardiology follow-up.

## Discussion

This case illustrates the high risk of infective endocarditis in adults with congenital heart disease (ACHD), even decades after surgical repair. Patients with bicuspid aortic valves and repaired coarctation remain vulnerable to late complications, including valvular dysfunction, aortic dissection, and endocarditis.^1–4^ Loss to follow-up in this patient removed opportunities for surveillance, counselling, and preventive measures such as antibiotic prophylaxis around invasive dental work. While National Institute for Health and Care Excellence guidance (CG64) no longer recommends universal dental prophylaxis,^5^ ESC guidelines continue to advocate it for selected high-risk groups, including those with prosthetic material or prior endocarditis.^3,6^

Several ‘red flags’ for endocarditis were overlooked: Persistent fever, embolic renal infarction, new conduction abnormalities, and rising inflammatory markers. Endocarditis should always be suspected in ACHD patients presenting with sepsis of uncertain origin.^7–9^ Point-of-care ultrasound in the emergency setting can accelerate recognition of valvular pathology and guide early referral.^10^ New conduction disturbance in aortic valve endocarditis should raise concern for perivalvular extension (aortic root abscess) and prompt urgent TOE and surgical review.^6^

Another important consideration is myocardial infarction secondary to septic embolism. This mechanism may account for the patient’s elevated troponins and new ST-segment changes, in addition to Type II myocardial injury from sepsis.^11^

Ultimately, delayed diagnosis stemmed from three system-level failures: dismissal of a positive blood culture, incomplete synthesis of multisystem warning signs, and absence of structured ACHD follow-up.^12,13^ Early echocardiography is emphasized by ESC guidelines in any septic patient with structural heart disease.^3,6^ In this case, only urgent surgery achieved a favourable outcome, underscoring the need for vigilance, structured transition programmes, and multidisciplinary management to improve survival.^1,14^

Permanent pacing was required for postoperative complete atrioventricular block. Given severe left ventricular systolic dysfunction was found (EF 20–30%), biventricular pacing (cardiac resynchronization therapy) was selected, consistent with pacing/CRT guideline recommendations.^15^

Strengths of this report include multimodality imaging and inclusion of the patient’s perspective to highlight system-level vulnerabilities. Limitations are inherent to a single case and do not allow causal inference regarding dental work or prophylaxis strategies.

### Patient perspective

As a child I was told my heart surgery had gone well and that I could live normally. I grew up knowing I had a slight murmur, stayed active as advised, and heard nothing further about adult follow-up. In 2010, a life-assurance assessment flagged a ‘heart condition’, with one insurer declining cover and another doubling premiums, suggesting annual checks. My GP reassured me this wasn’t necessary, and I accepted that. During the pandemic, I was offered early COVID and flu vaccines ‘because of my heart’, yet no one mentioned routine cardiac review or dental precautions.

The week I became unwell, an emergency GP remarked mine was one of the clearest murmurs he’d heard—a warning, in hindsight. I deteriorated quickly, reached ICU, and couldn’t relay my history. In recovery I asked why a loud murmur wasn’t a trigger; I was told there’s no standardized grading that reliably prompts action. I accept it wouldn’t have prevented endocarditis, but earlier recognition might have limited the damage.

Hospital and post-discharge care were excellent, and the ICU debrief was invaluable. Still, communication gaps persisted: I misunderstood my 6-month driving restriction (it related to my last VF, not the device), and routine queries were bounced between services. Going forward, clear information and accessible specialist contact are essential for confidence in recovery.

## Supplementary Material

ytag284_Supplementary_Data

## Data Availability

The data underlying this article are available within the article itself. No additional data are available.

## Lead author biography

Dr Ryan M. Wilson MBBS B Med Sci PG Cert Med Ed MRCP 1 is an SHO doctor at Frimley Health NHS Foundation Trust, UK. He has completed rotations in cardiology, respiratory medicine, and two in acute medicine, alongside a PG Cert in Medical Education. His academic work includes case reports on infective endocarditis and oncogenic osteomalacia, and multiple audits on echocardiography and service pathways. He has a strong interest in adult congenital heart disease, endocarditis, and health-system safety, with a long-term goal of a career in cardiology focused on quality improvement and education.

## Notes

**Consent:** Written informed consent for publication of this case report, including clinical data and images, was obtained from the patient in accordance with COPE guidelines.
