# Supplementary material for: Case report: life-threatening endocarditis in an adult with repaired congenital heart disease lost to follow-up
Source: Eur Heart J Case Rep. 2026 Apr 22;10(5):ytag284. doi: 10.1093/ehjcr/ytag284 (PMC13178788; doi:10.1093/ehjcr/ytag284)
Supplement: ytag284_Supplementary_Data [file ytag284_supplementary_data.zip › Suppl Video legends.docx]

| Video legend:   - Video 1. Diagnostic TOE. Moving image. Demonstration of a root abscess - Video 2. Diagnostic TOE. Moving image. Demonstration of left ventricular dilation. - Video 3. Diagnostic TOE. Moving image. Demonstration of the bicuspid aortic valve. - Video 4. Post-operative TTE. Moving image. Demonstration of aortic valve repair with colour doppler. |
| --- |
